# Impact of gut microbiota composition on black cutworm, *Agrotis ipsilon* (hufnagel) metabolic indices and pesticide degradation

**DOI:** 10.1186/s42523-023-00264-6

**Published:** 2023-09-15

**Authors:** Omnia Abdullah ElKraly, Mona Awad, Hassan Mohamed El-Saadany, Sameh E. Hassanein, Tahany Abd Elrahman, Sherif M. Elnagdy

**Affiliations:** 1 Botany and Microbiology Department, Faculty of Science, Cairo University, Gamma St, Giza, 12613 Giza Egypt; 2 Bio-insecticides Production Unit, Plant Protection Research Institute (PPRI), Agricultural Research Center (ARC), Ministry of Agriculture, Dokki, Giza Egypt; 3 Department of Economic Entomology and Pesticides, Faculty of Agriculture, Cairo University, Cairo, Egypt; 4 College of Biotechnology, Misr University for Science and Technology (MUST), Giza, Egypt

**Keywords:** *Agrotis ipsilon*, Endosymbionts, Gut bacteria, Insecticide degradation, Metabolism

## Abstract

Endosymbionts are known to have significant effects on their insect hosts, including nutrition, reproduction, and immunity. Insects gut microbiota is a critical component that affects their physiological and behavioral characteristics. The black cutworm (BCW), *Agrotis ipsilon*, is an economically important lepidopteran pest that has a diverse gut microbiome composed of nine species belonging to three phyla: Proteobacteria, Actinobacteria, and Firmicutes. This study was conducted to investigate the diversity of gut bacteria isolated from BCW larvae and moths and their effects on metabolism and pesticide degradation. The bacterial isolates were identified using the *16 S* rRNA gene. The study showed that the gut microbiome composition significantly affected the metabolism of BCW larvae. Based on the screening results of synthesis of digestive enzymes and pesticide degradation, *Brachybacterium conglomeratum* and *Glutamicibacter* sp were selected to perform the remaining experiments as single isolates and consortium. The consortium-fed larvae showed high metabolic indices compared to antibiotic-fed larvae and the control. The gut bacteria were also shown to degrade three pesticide groups. Concerns regarding the health risk of chlorpyrifos have been raised due to its extensive use in agriculture. The isolated *B. conglomeratum* was more effective in chlorpyrifos degradation than the consortium. Furthermore, the study also examined the presence of sex related endosymbionts (*Wolbachia*, *Spiroplasma*, and *Rickettsia*) in the reproductive tissues of adults. The outcomes demonstrated that none of the examined endosymbionts existed. In conclusion, the study highlights the importance of the gut microbiome in insect physiology and behavior and its potential applications in biotechnology. It provides insights into developing eco-friendly pest control and bioremediation strategies using gut bacteria.

## Introduction

Insects are the most diverse and abundant class of animals on earth, occupying nearly all terrestrial ecological niches, with about 53% of all living species being insects [91]. Lepidoptera, the second-largest order of insects, are easily recognizable in nature and have over 150,000 species with a wide range of morphologies and behaviors [66, 95]. While many insects are beneficial to plants, aiding in pollination, seed dissemination, and plant defense [60], butterflies and moths, which are crucial to ecosystems as pollinators and prey in the food chain, can also cause significant losses in agriculture due to their caterpillars’ feeding habits [106].

One such pest is *Agrotis ipsilon* (Hufnagel), commonly known as the black cutworm (BCW) (Lepidoptera: Noctuidae), which infests various crops worldwide. BCW caterpillars are polyphagous pests that feed on several commercially significant cereals and vegetables [82]. *A. ipsilon* is prevalent in Egypt and affects various field crops, vegetables, cotton, and turfgrasses [13, 67, 68]. The caterpillars feed by chopping off the leaflets and plant stems at the base during the early phases of plant development [2, 61].

The excessive usage of synthetic chemical pesticides to manage these pests has resulted in insecticide resistance and unintended deadly effects on non-target biota [44, 82]. Controlling BCW with insecticides is challenging due to insecticide resistance and the larvae’s nocturnal feeding habits [3, 38, 45]. One of the most widely used agricultural organophosphorus (OP) pesticides is chlorpyrifos (CP), which was field-tested in 1982 as a potential pesticide for the control of cutworm (*Agrotis orthogonia*) [58]. However, concerns have been raised regarding its health risks due to its extensive use in agriculture. This study focuses on designing and creating efficient methods for eliminating CP [101].

Effective control of insect pests requires the development of new eco-friendly and sustainable solutions. Using native microorganisms, particularly bacteria, has become a popular approach due to its effectiveness, affordability, and environmental friendliness [101]. Recently, the potential exploitation of insect symbionts has emerged as a promising tool for Integrated Pest Management (IPM) programs, made possible by understanding bacterial symbionts’ interaction with their insect hosts [44, 60]. Many symbiotic microorganisms participate in various relationships with insect hosts due to their high diversity and prolonged coexistence, and insects harbor bacterial endosymbionts that can profoundly affect their host’s biology [26, 91]. Endosymbionts can reside within insect cells or colonize the gut lumen, lining of insect cavities, and body surface, and can be classified as intra- and extracellular [27, 39, 95].

The insect gut is a “hot spot” for various microbial activities, illustrating a range of microbial connections from pathogenicity to obligate mutualism [25]. Most insects have a diverse and complex microbial population in their gut, many of which are crucial for growth, development, immunity, digestion, feeding, defense against pesticides, and defense against poisonous plant secondary metabolites. These tasks are accomplished through a range of enzymes that the bacteria create, including breakdown of harmful compounds, amino acid synthesis, and carbohydrate usage [27, 39, 48, 103]. The endosymbiotic relationship between insects and gut bacteria has developed due to numerous essential microbial functions, such as the production of enzymes, detoxification of insecticides and plant defense compounds, maintenance of the life cycle, host fertility, bioremediation, pest biocontrol, production of antimicrobial compounds, and provision of vitamins, amino acids, and lactic acids to their hosts [25, 29, 30, 33, 78, 93, 98, 103]. Insects have evolved symbiotic associations with various microbes (bacteria) for nutritional benefits, such as the digestion of food components, through the manufacture of several important hydrolytic enzymes, such as amylase, cellulase, lignocellulase, protease, lipase, xylanase, pectinase, chitinase, laccase, etc. [5].

Understanding host-microbe interactions can be applied for biotechnological purposes in two ways: either by using symbiotic interactions to control agricultural pests or vector-borne diseases or to improve the health of economically significant insects like honeybees, or by applying symbiont-produced substances like small bioactive molecules or enzymes for pharmaceutical use or industrial processes [14, 94, 106].

Chen et al. [19] reported that certain lepidopterans are associated with complex consortia of bacteria that may play a crucial role in metabolic resistance. In nature, microorganisms often coexist in consortia, which are groups of two or more interacting microbial populations that occur in various environmental niches. However, naturally occurring microbial consortia have several limitations that hinder their practical applicability in biotechnology, including difficulties in cultivation, lengthy operating cycles, low conversion efficiency, and poor stability and controllability [52, 62]. Synthetic microbial consortia, on the other hand, have been shown to carry out even more challenging tasks and withstand more variable environments than monocultures [17, 32], making them a promising frontier in synthetic biology. Widder et al. [99] reported on the multicellular mechanisms that govern cell-cell interactions in consortia, including commensalism, amensalism, mutualism, parasitism, and parasitism leading to predation.

Microbial resources, whether used individually or in groups, have the potential to significantly reduce pesticide toxicity. Kumar et al. [52] found that members of the phyla Actinobacteria, Ascomycota, Bacteroidetes, Basidiomycota, Chlorophyta, Cyanobacteria, Firmicutes, and Proteobacteria were the best sources for breaking down several types of pesticides, including Carbamates, Organochlorines, Organophosphates, and Pyrethroids. They also identified several microorganisms, such as *Arthrobacter, Aspergillus, Bacillus, Burkholderia, Chlamydomonas, Methylobacterium, Nocardioides, Nostoc, Phanerochaete, Pseudomonas, Sphingobacterium, Sphingomonas*, and *Trichoderma*, that can break down various pesticides.

The objective of this study was to isolate BCW gut bacteria with unique abilities, investigate their impact on host metabolism, and explore potential applications in biotechnology and pesticide degradation as a single isolate and consortium. The study aimed to (1) characterize the composition and diversity of bacterial gut communities in BCW adults and larvae, (2) evaluate the degradation ability of all bacterial isolates for different carbon sources and pesticides, (3) examine the impact of gut bacteria on BCW larval nutrition metabolism, and (4) investigate the ability of gut bacterial isolates to degrade pesticides to protect their host and contribute to pesticide resistance.

## Materials and methods

### Insect sampling and rearing

*A. ipsilon* larvae were collected from a bean field (*Vicia faba* L.) in Ismailia, Egypt, and were subsequently mass-reared in the laboratory on fresh castor oil bean leaves (*Ricinus communis* L.) under controlled conditions of 27 ± 2 °C, 60–80% RH, and a 16 − 8 light-dark cycle. The emerged moths were provided with a 20% sugar solution [61, 64] for feeding. The identification of *A. ipsilon* larvae and moth samples was performed using a morphological key [9].

### Bacterial isolation and identification

Nine healthy and active *A. ipsilon* larvae from the fourth larval instar and eighteen moths (9 male; 9 female) were selected and washed in tap water. The specimens were immobilized on ice for approximately five minutes before being sterilized, and the wings of the moths were removed. All specimens underwent a two-minute surface sterilization in 70% ethanol, followed by rinsing in sterile distilled water. Under sterile conditions and a stereomicroscope, intact guts were dissected from the specimens and placed in a sterile Petri dish containing sterile saline solution (0.85% NaCl) [15, 34, 71]. Sterile normal saline (0.85%) has been recommended to assist in maintaining bacterial cell integrity and viability [12, 90]. For each three guts, the samples were homogenized and vortexed in 2 mL centrifuge tubes with 1 mL of saline solution to eliminate the microbial cells from the gut wall. To isolate slow-growing bacteria, the gut homogenate was serially diluted in sterile saline (10^4^ to 10^6^), and then triplicate pour plates were prepared on nutrient agar (NA) media. The process was carried out aerobically at 30 °C for 7 days, with daily observations. Ten single colonies were selected and purified based on their color, size, and morphology from the NA plates. The pure single colonies were kept in glycerol for preservation until they were ready to be identified and tested for different activities [36, 43, 65, 74].

### Molecular identification of bacterial isolates

#### Bacterial genomic DNA extraction

DNA was extracted from the bacterial isolates using GeneJET Genomic DNA Purification Kits #K0701 and #K0702 (Thermo Scientific, Waltham, MA, USA) following the manufacturer’s instructions. A single colony was used to infect 5 mL of nutrient broth (NB), and the mixture was incubated for 24 h at 37 °C with shaking at 150 rpm. The extracted DNA was visualized after gel electrophoresis and stored at -20 °C.

#### Amplification of bacterial *16 S* rRNA

The bacterial isolates were identified based on their *16 S* rRNA gene sequence. The genomic DNA template was used to amplify approximately 123 base pairs of the *16 S* rRNA gene using the Bact1369F (5’CGGTGAATACGTTCYCGG3) and Prok1492R (5’GGWTACCTTGTTACGACTT3) primers [4, 84, 92]. PCR reactions were performed using amaR OnePCR Master Mix (GeneDireX, Taiwan, China) and 10 pmol of each primer. The thermocycling conditions were as follows: initial denaturation for 5 min at 95 °C, denaturation for 20 s at 95 °C (35 cycles), annealing for 30 s at 50 °C, extension for 30 s at 72 °C, and final extension for 5 min at 72 °C. The amplicons were purified using GeneJET PCR purification kits #K0701 and #K0702 (Thermo Scientific, Waltham, MA, USA), and Sanger sequenced from both directions using the Bact1369 forward and Prok1492 reverse primers. The *16 S* rRNA gene sequences were assembled using DNA Baser assembler v5.15 (Romania). Taxonomy was assigned using BLAST against the NCBI database based on the top and the more frequent BLAST hit. The partial *16 S* rRNA gene sequences for isolated bacteria have been submitted to the NCBI under accession numbers (Table 1).

**Table 1:** Screening of bacterial isolates activity in vitro for (digestive) enzymes production and ability to grow and degrade different pesticides (100 ppm). The numbers represent the average values of the clear zone indices; however, the signs (-, +, and ++) denote whether there has been no growth, growth, or good growth of bacterial colonies in media supplemented with pesticides

Isolates	GenBank accession number(s)	The mean of degradation enzymes clear zone indices.						Growth and the mean of clear zone index in media supplemented with pesticides (100 ppm)		
		CMC	Xylan	pectin	Starch	Tween 80	Gelatin	Chlorpyrifos	Emamectin benzoate	Lambda-cyhalothrin
*Mammaliicoccus sciuri* (*Staphylococcus sciuri* )	OP023879	1.2	0	0	3	0	1.1	-	+	+
*Brachybacterium conglomeratum*	OP023880	2	1.3	1.2	5	2	1.2	++ (1.2)	++ (1.2)	+
*Klebsiella variicola*	OP023881	1.3	0	1.1	5	1.2	0	-	++ (1.3)	++ (1.1)
*Corynebacterium casei*	OP023882	0	1.1	1.1	2	3	0	-	+	++ (1.2)
*Glutamicibacter* sp.	OP023884	2	1.2	1.3	4	1.5	1.3	++ (1.1)	++ (1.1)	++ (1.1)
*Morganella morganii*	OP023889	2	0	0	3	1.5	1.1	-	+	+
*Klebsiella oxytoca* strain 14	OP023890	1.2	1.1	1.2	1.3	2	0	+	++ (1.1)	++ (1.1)
*Klebsiella oxytoca* strain 15	OP023891	1.4	1.2	1.2	3	2	0	+	++ (1.2)	++ (1.1)
*Enterobacter* sp.	OP023892	0	0	1.1	3	2	1.1	+	++ (1.2)	-
*Bacillus subtilis*	OP023893	1.5	0	1.2	3	1.4	1.2	-	+	-

The multiple sequence alignment (MSA) was performed using the online tool MUSCLE (stands for **MU**ltiple **S**equence **C**omparison by **L**og- **E**xpectation). MUSCLE is claimed to achieve both better average accuracy and better speed than ClustalW2. (https://www.ebi.ac.uk/Tools/msa/muscle/ (accessed on 28 March 2023)). The phylogenetic tree was generated by the maximum likelihood method using Mega 11 and visualized by FigTree (http://tree.bio.ed.ac.uk/software/figtree/ (accessed on 28 March 2023)).

#### Screening for sex related endosymbiotic Bacteria

Ten samples of BCW (7 female and 3 male) were sterilized with 75% alcohol and rinsed twice with sterile water before DNA extraction. Abdomen parts of male and female BCW moths were dissected aseptically using sterilized forceps and scalpels and placed in an eppendorf for DNA extraction. Specific primers were used to screen for sex related endosymbionts (*Wolbachia*, *Spiroplasma*, and *Rickettsia*) (Table 2). The PCR cycle consisted of an initial denaturation step at 94 °C for 1 min, 35 cycles of denaturation at 94 °C for 30 s, annealing at 58 °C for 45 s, and extension at 72 °C for 1 min, followed by a final extension step at 72 °C for 10 min. PCR products were visualized by running on a 2% TAE-agarose gel stained with ethidium bromide [7, 79].

**Table 2:** The primer names, target organism, amplified gene, sequences, annealing temperatures, and references are listed for each COI and endosymbiotic primer pair that was used in our research

Organism	Gene	Primer Name	Sequence	Annealing temperature	Reference
BCW	mtDNA *COI*	RF_450F	5`-ACCTGATATGGCTTTTCCCCG-3`	58 ° C	[79]
		RF_1123R	5`-ACCAAGAATTCCAAAGGTTTCTTT-3`		
all Eubacteria	*16 S* rRNA	Bact1369F	5`- CGGTGAATACGTTCYCGG-3`	50 °C	[92].
		Prok1492R	5`- GGWTACCTTGTTACGACTT-3`		
*Wolbachia*	*Wsp*	wsp_F1	5`-GTCCAATARSTGATGARGAAAC- 3`	56 °C	[10].
		wsp_R1	5`-CYGCACCAAYAGYRCTRTAAA − 3`		
*Rickettsia*	*gltA*	RicF141	5’-TCGGTTCTCTTTCGGCATTTTA-3’	56 °C	[35].
		RicR548	5’ -GCATATTTATCACCGCTTCATT-3’		
*Spiroplasma*	*23 S* rDNA	SP-ITS-JO4	5’-GCCAGAAGTCAGTGTCCTA ACCG-3’	56 °C	[57].
		SP-ITS-N55	5’-ATTCCAAGCCATCC ACCATACG-3’		

### Diversity indices of bacterial gut isolates

To estimate the relative frequency of bacterial gut isolates, the total number of isolates from a moth or larval intestine was divided by the total number of taxa [36]. The following diversity indices were calculated: Shannon’s index [83], Simpson’s index [40], richness [59], and evenness [49].

### Screening of isolates for enzyme production

#### Screening for digestive enzyme production

##### Cellulase, Xylanase, Pectinase, and Amylase

To test the ability of gut bacterial isolates to produce enzymes, they were streaked on Berg’s agar media and cultured aerobically for 7 days at 30 °C. Berg’s agar medium (Berg et al., 1972) was used without changing the minimal medium’s composition and supplemented with carbohydrate substrates (0.1% carboxymethylcellulose (CMC), 1% oat spelled xylan, 1% pectin, and 1% starch) in appropriate plates (n = 3). The clear zone around the colonies was measured by flooding the plates with 0.2 g/l potassium iodide for five minutes, and the index was calculated using the ratio of the clear zone diameter to the bacterial colony diameter [5, 23, 72].

##### Lipolytic enzymes

Isolates were inoculated in triplicates in Tween 80 medium [87] containing peptone, NaCl, CaCl_2_.2H_2_O, agar, and Tween 80 (g/L). The medium was incubated at 30 °C for 7 days [51, 100], and the appearance of a visible precipitation zone surrounding the colony served as an indicator of success. The index was determined as previously described.

##### Protease enzymes

Isolated bacterial strains were inoculated on agar media containing gelatin and agar (10 g/l gelatin and 20 g/l agar) to test for the production of protease enzymes in triplicates. A hydrolysis zone was observed after three days of incubation at 30 °C for the inoculation plates [23].

#### Screening of pesticide-degrading bacteria

In vitro investigations were conducted on three distinct pesticide groups (pyrethroid (lambda-cyhalothrin), organophosphate (chlorpyrifos ethyl), and natural (emamectin benzoate)). All isolates were streaked on 1/10 diluted nutrient agar supplemented with 100 ppm of each pesticide and cultured for 7 days at 30 °C [20]. Isolates that can grow or degrade pesticides and form a clear zone index were detected as previously mentioned in Sect. 2.5.

### Effects of antibiotics and gut bacteria on the nutrition indices of *A. ipsilon*

From the screening results, two bacterial isolates, *Brachybacterium conglomeratum* and *Glutamicibacter* sp., were selected to complete the experiments as single isolates and in a consortium. The Cross Streak technique was used to assess the synergistic activity between the two isolates. Each isolate was streaked on NA media in a straight line at a 90° angle, then incubated for 5 days at 30 °C. The absence of an inhibition zone at the intersection of streaking lines of colonies was used to analyze the synergistic growth interactions among the tested isolates. The selected bacterial isolates (*Glutamicibacter* sp., *B. conglomeratum*, or in consortium) were adjusted to a density of 0.5 McFarland (1.5 × 10^8^ CFU/mL).

#### Treatment groups

The *(A) ipsilon* eggs were divided into two groups: gnotobiotic obtained by dechorionizing the eggs, and the other group was washed with distilled water. Overall, there were three treatments: gnotobiotic + castor leaves with antibiotics (GFA), non-dechorionated eggs + castor leaves with bacteria (BF) (*Glutamicibacter* sp. only, *(B) conglomeratum* only, and in consortium), and non-dechorionated eggs + castor leaves without additives as control (CF).

##### The gnotobiotic fed antibiotics (GFA) larvae rearing method

Newly hatched *A. ipsilon* larvae were fed castor leaves soaked in an antibiotic cocktail for half an hour for seven days to examine the antibiotic cocktail efficiency. The gut bacteria were isolated on NA plates for two days at 30 °C to examine the antibiotic efficiency on the count and diversity of gut bacteria compared with sugarcane feeding larvae as control. The *A. ipsilon* eggs for the GFA group were surface sterilized before hatching to prepare GF larvae. Castor leaves were soaked in the antibiotic solution for 30 min at a final concentration of 600 mg/L of kanamycin, tetracycline, gentamicin, and erythromycin to disrupt the normal structure of the black cutworm’s gut microbiome.

##### The BF rearing method

The two groups of non-dechorionated eggs were put in a nylon mesh bag and given a distilled water wash. The chosen gut isolates were cultured overnight in Luria-Bertani (LB) broth medium at 30 °C with shaking, and castor leaves were soaked in different bacterial solutions with the same concentration for 30 min.

##### Control group

Conventional castor leaves soaked in sterile distilled water were used in rearing the control larvae.

#### Nutritional analysis

The treatment was carried out until the larvae reached the 4th instar, after which their hemolymph was extracted to analyze metabolic indices such as glucose, protein, and triglyceride (TAG) concentrations. Prior to hemolymph collection, larvae were washed under running water to eliminate excrement and food particles, followed by surface sterilization with 70% ethanol and immobilized for 2–3 min on ice. Hemolymph was collected from the last larval prolegs’ epidermis using a fine sharp needle, and about 0.5 ml of hemolymph was collected in labeled 1.5 ml clean microcentrifuge tubes. To inhibit hemolymph melanization, 2 µl 0.2% phenylthiourea (PTU) was added to each tube [1, 65]. The glucose, protein, and TAG concentrations were determined using the Bio-diagnostic protein Biuret method (colorimetric method, Giza, Egypt), glucose Measurement Kit (enzymatic colorimetric method, Giza, Egypt), and Triglyceride Assay Kit (enzymatic colorimetric method, Giza, Egypt), respectively. Each treatment group consisted of 10 larvae. At the end of the treatment, some larvae were selected for gut bacteria isolation and counting, as described in Sect. 2.2.

### Chlorpyrifos degradation assay

As one of the banned pesticides, chlorpyrifos was selected for the bacterial biodegradation experiment. The selected bacterial isolates (*Glutamicibacter* sp., *B. conglomeratum*, or in consortium) were adjusted to a density of 0.5 McFarland (1.5 × 10^8^ CFU/mL) and grown in chlorpyrifos minimal salt liquid medium. The medium contained 0.7 g/L monopotassium phosphate, 0.9 g/L sodium hydrogen phosphate, 2 g/L sodium nitrate, 0.4 g/L magnesium sulfate heptahydrate, 0.1 g/L calcium chloride dehydrate, 0.004 g/L ferrous sulfate heptahydrate, 0.003 g/L manganese sulfate monohydrate, and 0.0012 g/L ammonium molybdate tetrahydrate, with a pH of 6.7 ± 0.2. After 4 and 10 days of incubation on a rotary shaker at 150 rpm and 35 °C, samples were collected to determine the pesticide content. Additionally, samples were re-cultured on NA plates to confirm bacterial viability [20].

Chlorpyrifos was extracted from samples using the QuEChERS method [6]. Each sample was weighed at about 10 g then 10 mL acetonitrile was added, and the mixture was agitated for one minute. The mixture was then agitated rapidly for one minute and centrifuged at 4000 rpm for five minutes. Next, 4.0 g of MgSO_4_ anhydrous, 1.0 g of NaCl, 1.0 g of trisodium citrate dehydrate, and 0.5 g of disodium hydrogen citrate sesquihydrate were added. Pesticide reference standards were purchased from Dr Ehrenstorfer (Augsburg, Germany) with purities > 95% [41, 77].

The pesticide concentration was determined using an Agilent HPLC system with the following chromatographic conditions: Agilent HPLC 1260 infinity 11 autosamplers, DAD detector, wavelength: 200 nm, column temperature: 30 °C, *Ascentis apelco*, C18 column (150 × 4.6 mm, 15 μm), mobile phase: Acetonitrile: methanol (90:10), flow rate: 0.6 mL/min, and injector volume: 20 µl.

### Statistical analysis

In this study, all statistical analyses were conducted using Proc ANOVA in SAS, with significant differences between means compared at P = 0.05. To analyze the nutrition metabolism indices, including larvae body weight, glucose, protein, and TAG concentrations, a one-way analysis of variance (ANOVA) was performed with three treatment groups (GFA, BF, and CF) and different exposure times. Similarly, a separate one-way ANOVA was used to analyze the diversity indices. The gut bacteria counts were expressed as colony-forming units per BCW gut (CFU/gut), and count results were log^10^ transformed to normalize the data before statistical analysis.

## Results

### Bacterial isolation, identification, and diversity

The mean gut bacterial counts in larvae ranged from 1.2 × 10^7^ to 1.4 × 10^7^ CFU gut^− 1^, while the counts in female and male moths ranged from 1.5 × 10^7^ to 1.8 × 10^7^ CFU gut^− 1^. Statistical analysis indicated no significant differences between the counts in both stages (P = 0.182) and gender (P = 0.185). A total of 53 isolates were obtained from 3 larval guts and 6 moth guts of BCW under aerobic culture conditions. These isolates were identified by *16 S* rRNA gene sequence analysis and assigned to eight genera and nine species belonging to three phyla: Proteobacteria (56.6% of total bacteria), Actinobacteria (24.5%), and Firmicutes (18.9%) (Table 3).

**Table 3:** Taxonomic positioning, distribution, and frequency (%) of gut bacterial isolates at the phylum, genus, and species level isolated from the gut of *Agrotis ipsilon.* L = larvae, F = female moth, and M = male moth

Phylum	Family	Genera	Species	Number (n = 53)	Distribution	Frequency (%)
Proteobacteria (56.6%)	Enterobacteriaceae (41.5%)	*Klebsiella* (28.3%)	*Klebsiella oxytoca*	11	L, F, M	20.8
			*Klebsiella variicola*	4	F, M	7.5
		*Enterobacter*	*Enterobacter* sp.	7	L, F, M	13.2
	Morganellaceae	*Morganella*	*Morganella morganii*	8	F, M	15.1
Actinobacteria (24.5%)	*Micrococcaceae*	*Glutamicibacter*	*Glutamicibacter* sp.	8	L, F, M	15.1
	Dermabacteraceae	*Brachybacterium*	*Brachybacterium paraconglomeratum*	4	L, F	7.5
	Corynebacteriaceae	*Corynebacterium*	*Corynebacterium casei*	1	L	1.9
Firmicutes (18.9%)	Bacillaceae	*Bacillus*	*Bacillus subtilis*	7	L, F, M	13.2
	Staphylococcaceae	*Staphylococcus*	*Mammaliicoccus sciuri* (*Staphylococcus sciuri*)	3	L	5.7

**Fig. 1:**
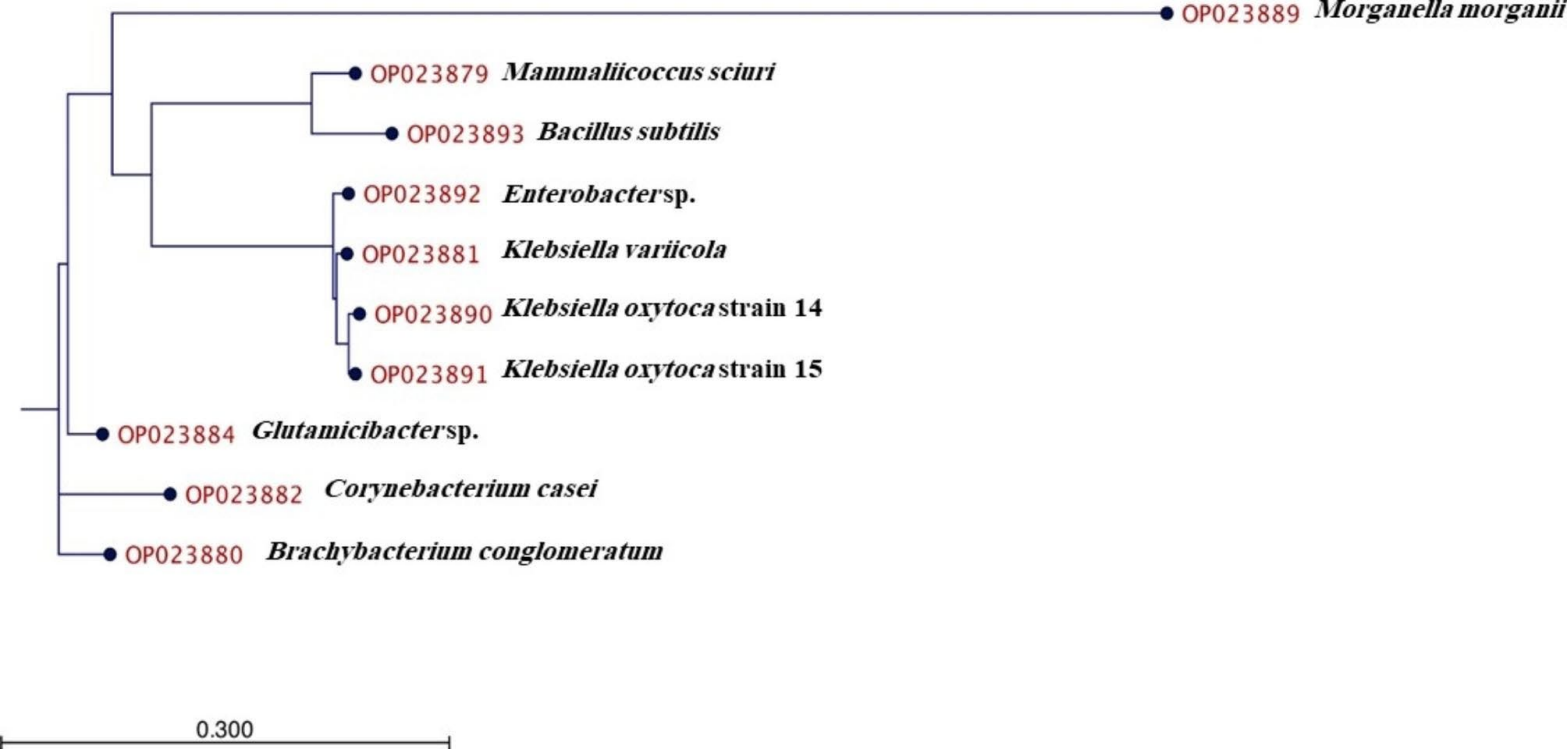
The phylogenetic tree for *A. ipsilon* gut bacteria based on *16 S* rRNA gene sequences. The accession numbers were identified as OP023879: *Mammaliicoccus sciuri*, OP023880: *Brachybacterium conglomeratum*, OP023881: *Klebsiella variicola*, OP023882: *Corynebacterium casei*, OP023884: *Glutamicibacter* sp., OP023889: *Morganella morganii*, OP023890: *Klebsiella oxytoca* strain 14, OP023891: *Klebsiella oxytoca* strain 15, OP023892: *Enterobacter* sp., and OP023893: *Bacillus subtilis*

Based on the constructed phylogenetic tree (Fig. 1), The evolutionary distance (substitutions per sequence site) between taxa indicated that the accessions OP023890 and OP023891 (*Klebsiella oxytoca* strain 14 and *Klebsiella oxytoca* strain 15 respectively) are the more closely related sequences. Also, accessions OP023890, OP023891, OP023881 and OP023892 (*Klebsiella oxytoca* strain 14, *Klebsiella oxytoca* strain 15, *Klebsiella variicola* and *Klebsiella variicola*) are within the same group that all sequences belong to Enterobacteriaceae family. While OP023882 (*Corynebacterium casei*) and OP023880 (*Brachybacterium conglomeratum*) are the most diverse sequences which belongs to the most diverse bacterial families (Corynebacteriaceae and Dermabacteraceae respectively).

Table (2) shows the distribution of gut bacterial isolates in larvae and moth (female and male) of black cutworm. The richness, evenness, and diversity indices of gut bacterial isolates in female and male moths were not significantly different (P = 0.46579) but were more diverse than in larvae non-significantly (P = 0.718516) (Table 4). The most prominent genus across the tested life stages was *Klebsiella* (28.3%). Two strains of *Klebsiella oxytoca* (*K. oxytoca* strain 14 and *K. oxytoca* strain 15) were the most dominant. Also, *Glutamicibacter* sp., *Morganella morganii*, *Bacillus subtilis*, and *Enterobacter* sp. were stably represented in the gut of BCW through the tested life stages. *Corynebacterium casei* was the least represented (1.9%).

**Table 4:** Taxonomic diversity indices of the *Agrotis ipsilon* bacterial gut in moth and larvae

Characters	Moth			Larvae
	Female	Male	collective	
Number of isolates	17	14	31	22
Menhinick’s richness index	1.46	1.34	1.08	1.49
Evenness	0.78	0.91	0.67	0.823
Shannon’s diversity index	1.38	1.47	1.19	1.6
Simpson’s diversity index	4.69	5.06	4.39	8.25

The tested bacteria did not colonize the reproductive tissues of the studied *A. ipsilon* moths according to the sex related endosymbionts (*Wolbachia*, *Spiroplasma*, and *Rickettsia*).

### Screening of isolates

#### Screening of isolates for digestive enzyme production

**Fig. 2:**
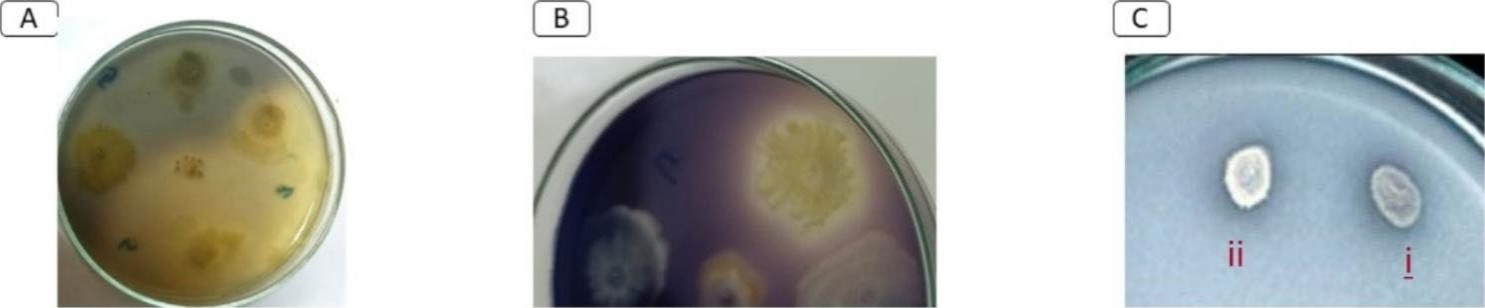
Activities of RPW gut isolates, (**A**) cellulase activity, indicated by the visualization of clear halos around the colonies; (**B**) amilolytic activity, indicated by the visualization of clear halos around the colonies; (**C**) chlorpyrifos degradation, indicated by the growth and clearance of the media surrounding the colony of *B. conglomeratum* (i) and *Glutamicibacter* sp. (ii)

Of the ten identified isolates, eight were able to degrade CMC, six were able to degrade xylan, seven were able to degrade pectin, and all were able to degrade starch. The clear zone indices are shown in Table 1; Fig. 2. Both *B. conglomeratum*, *Glutamicibacter* sp., and *M. morganii* were able to degrade CMC with a higher clear zone index (2) than *B. subtilis* (index of 1.5). *B. conglomeratum* could degrade both xylan and starch with high indices of 1.3 and 5, respectively. *Glutamicibacter* sp. achieved the highest clear zone index in pectinase production and also produced the highest index of degrading gelatin. *Corynebacterium casei* formed the largest precipitation zone index in Tween 80 medium.

#### Screening of pesticide-degrading bacteria

Table 1; Fig. 2 reveal that only *B. conglomeratum* and *Glutamicibacter* sp. isolates could grow and degrade chlorpyrifos. However, the two strains of *K. oxytoca* and *Enterobacter* sp. grew weakly in chlorpyrifos medium but did not form a clear zone. All isolates were able to grow in both emamectin benzoate medium and lambda-cyhalothrin medium, except for *Enterobacter* sp. and *B. subtilis*. *K. variicola* and both *K. oxytoca* strains efficiently degraded emamectin benzoate and formed a clear zone in lambda-cyhalothrin media, but *C. casei* exhibited the highest index.

*B. conglomeratum* and *Glutamicibacter* sp. produced all tested enzymes to degrade different polysaccharides, especially cellulase, protease, and lipolytic enzymes. Hence, they were selected for the in vivo study on the nutrition indices of BCW larvae, as well as to assay bacterial biodegradation of chlorpyrifos.

### Effects of antibiotics and gut bacteria on the nutrition indices of *A. ipsilon*

**Fig. 3:**
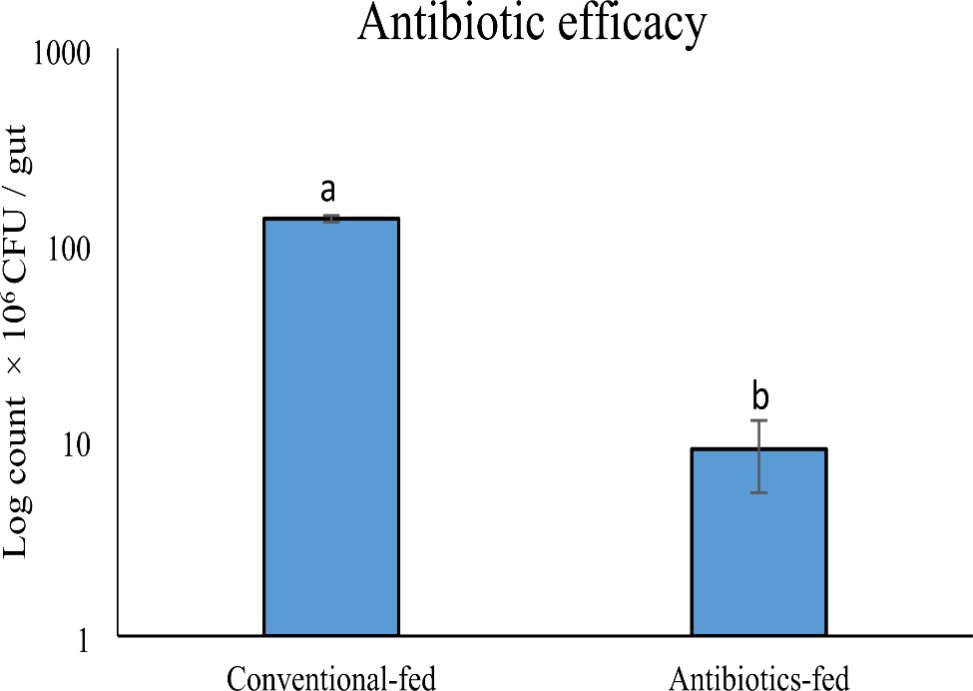
Gut bacterial count of the *A. ipsilon* larvae feeding on antibiotics cocktail compared to the control

Every day, the cross-streaked colonies were checked for inhibitory zones, but none were observed. It can be concluded that both isolates interacted synergistically. The efficiency of the antibiotic cocktail demonstrated a highly significant difference in gut bacterial count between CF and GFA larvae (Fig. 3). In addition, the antibiotic cocktail reduced bacterial diversity.

**Fig. 4:**
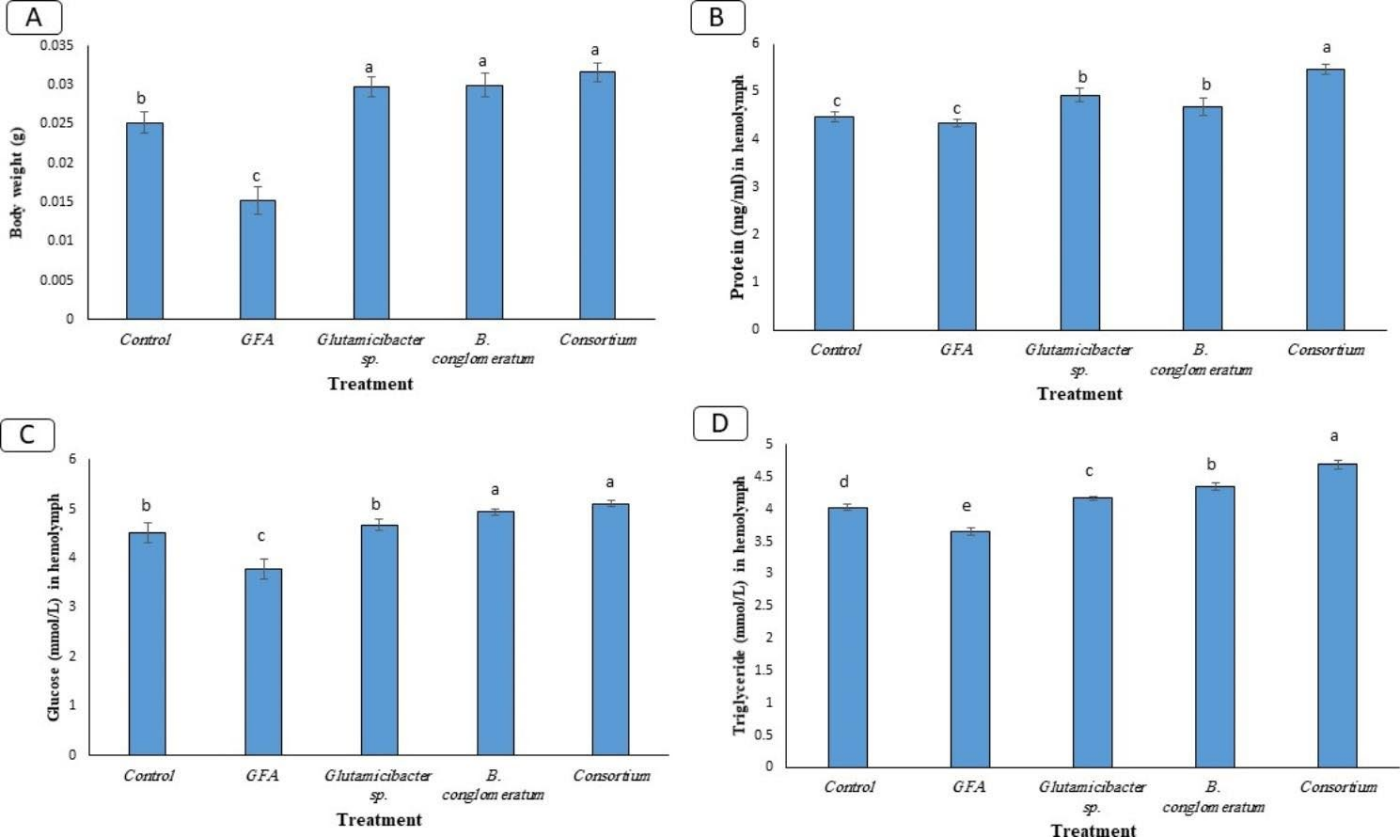
Impact of gut bacterial community structure in *A. ipsilon* larvae after feeding bacterial isolates and its consortium and antibiotics on the body weight (**A**) and the concentration of hemolymph protein (**B**), glucose (**C**), and triglyceride (**D**). The letters above the graphs indicate the statistical significance between groups (p < 0.05). Similar letters indicate no significant difference (P > 0.05)

**Fig. 5:**
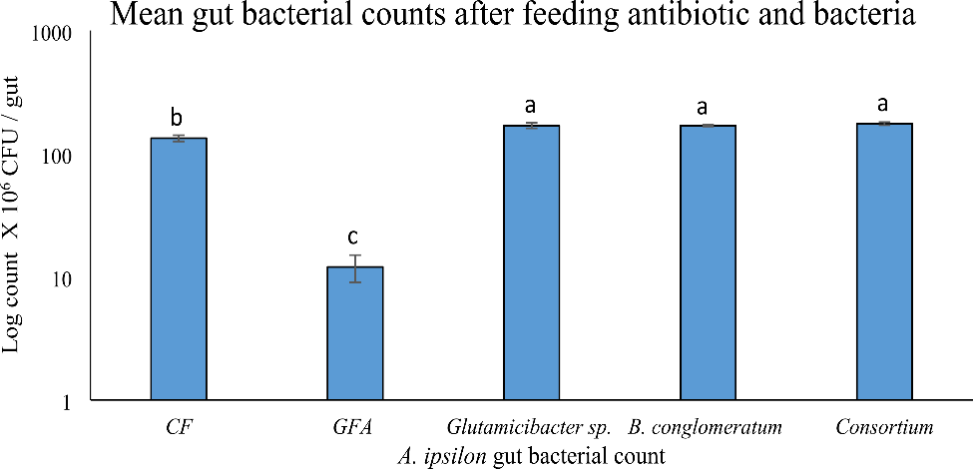
The gut bacterial count of the treated groups after feeding bacterial isolates and its consortium and antibiotics in *A. ipsilon* larvae. The letters above the graphs indicate the statistical significance between groups (p < 0.05). Similar letters indicate no significant difference (P > 0.05)

This study examined the impact of gut bacteria on the metabolism of the host by comparing nutrition indices, larval body weight, protein, glucose, and TAG concentrations in hemolymph among CF, GFA, and BF treatments (Fig. 4; Table 5). The net weight gain of bacterial-feeding larvae, both single isolates and consortium, was significantly higher than that of CF larvae. However, GFA cutworm larvae showed a significant decrease in weight gain (Fig. 4A). Hemolymph protein concentration decreased significantly in GFA larvae compared to CF, while it increased remarkably in BF larvae fed with single bacterial isolates and consortium (Fig. 4B). Hemolymph glucose concentrations were considerably higher in both consortium and *B. conglomeratum* isolate-fed larvae than in CF larvae but low in the GFA larvae group (Fig. 4C). However, TAG concentration in hemolymph showed a considerable difference in each treatment, with the highest observed in the consortium-fed larvae followed by *B. conglomeratum* and the lowest in GFA larvae (Fig. 4D). The gut bacterial count varied significantly between the feeding larvae groups (Table 6; Fig. 5) at the end of the experiment.

**Table 5:** Effect of *B. conglomeratum, Glutamicibacter* sp., and its consortium on the metabolism of chlorpyrifos against control in vitro in 4 and 10 days

Name	Exposure time (days)	Amount recovered (ppm)	Amount recovered (%)	Loss (%)
***B. conglomeratum***	Zero time	15	100	0
	After 4 days	5.2	39.88	60.12
	After 10 days	0.0	0.0	**100**
***Glutamicibacter*** **sp.**	Zero time	15	100	0
	After\ 4 days	13.01	99.77	0.03
	After 10 days	10.3	82.4	17.6
Consortium	Zero time	15	100	0
	After 4 days	9.1	69.78	30.31
	After 10 days	1.04	8.63	91.37
Control	Zero time	15	100	0
	After 4 days	13.04	100	0
	After 10 days	12.05	100	0

**Table 6:** One-way analysis of variance (ANOVA) with three treatment groups: gnotobiotic fed antibiotics (GFA), bacterial fed (BF) (*Glutamicibacter sp., B. conglomeratum*, or in consortium) and conventional fed (CF) as control (GFA, BF, and CF). Means with the same letter within each factor and character are not significantly different (P > 0.05)

Treatment	Weight (g)	Protein (mg/ml)	Glucose (mmol/L)	Triglyceride (mmol/L)	Bacterial count CFU/gut ×10^6^
**gnotobiotic fed antibiotics (GFA)**	0.01524^c^	4.343^c^	3.79^c^	3.65838^e^	12^c^
***Glutamicibacter*** **sp.**	0.0298^a^	4.92^b^	4.68^b^	4.16977^c^	169^a^
***B. conglomeratum***	0.03^a^	4.71^b^	4.94^a^	4.3524^b^	168.7^a^
**Consortium**	0.032^a^	5.47^a^	5.11^a^	4.69192^a^	175^a^
**Control (CF)**	0.0252^b^	4.471^c^	4.523^b^	4.02459^d^	133.3^b^
**F**	**88.49**	**101.58**	**59.55**	**262.24**	**416.68**
**P**	**< 0.001**	**< 0.001**	**< 0.001**	**< 0.001**	**< 0.001**
**LSD**	**0.0021**	**2.13145**	**0.1931**	**0.0699**	**10.629**

### Chlorpyrifos biodegradation assay

Both *B. conglomeratum* and *Glutamicibacter* sp. isolates, as well as their consortium, were able to grow on a minimal medium supplemented with 15 ppm chlorpyrifos. After 10 days of incubation, the CFU/mL count increased to 3.2 × 10^8^, 2.7 × 10^8^, and 3.4 × 10^8^ for *B. conglomeratum*, *Glutamicibacter* sp., and the consortium, respectively. The degradation of chlorpyrifos was quantified using the HPLC method. *B. conglomeratum* showed a high degradation rate, breaking down 60.12% and 100% of chlorpyrifos after 4 and 10 days of incubation, respectively, compared to the control (Table 5). In contrast, *Glutamicibacter* sp. exhibited weaker degradation, breaking down only 17.6% of chlorpyrifos after 10 days of incubation. Surprisingly, the consortium showed reduced degradation efficiency, breaking down only 91.37% of chlorpyrifos after 10 days, suggesting an antagonistic interaction between the two isolates during chlorpyrifos degradation.

## Discussion

In the realm of insect-microbe symbiosis, a captivating interplay unfolds as insects and their microbial partners establish dynamic relationships through coevolution. Insects offer stable habitats and essential nutrients, while microbes reciprocate with defensive strategies and nutritional support [104]. This collaboration is especially crucial in addressing insect pests, known for their ability to metabolize toxins and explore alternate nutrients. This motivated our focus on Lepidoptera’s enigmatic microbiota, encompassing various agricultural pests [19, 24].

Silkworms, for instance, provide a case in point, where gut microbial alterations impact circulating metabolites in the hemolymph. Key player *Bacillus subtilis* produces metabolites including B vitamins and antibacterial compounds, enhancing disease resistance and micronutrient availability [54]. Building on this, our study explores the intricate microbiota of the black cutworm (BCW), a significant crop pest, revealing taxonomic insights and functional enzyme potential.

The taxonomic analysis of BCW’s gut microbiota confirms established phyla like Proteobacteria, Actinobacteria, and Firmicutes, aligning with prior investigations [26, 27, 56, 60, 85, 88, 104]. Notably, the Enterobacteriaceae family’s persistent dominance, associated with polysaccharide degradation, underscores its vital functional role across developmental stages [91]. Our analysis identifies eight genera within the lepidopteran gut, echoing earlier findings [5, 16, 43, 81, 88, 97, 103, 106, 107]. An intriguing discovery is the stability of diversity indices, challenging previous observations of variance in larvae and adults’ feeding habits [91, 107]., enriching our understanding of BCW’s microbiota dynamics.

While prior studies highlighted reproductive tissue colonization by endosymbionts in Lepidoptera [47, 95], our research unveils the absence of sex-related endosymbionts within *A. ipsilon* reproductive population.

Our investigation spotlights the enzymatic potential within BCW gut microbiota. Synthesis of enzymes like cellulases, hemicellulases, xylanases, and more underpins nutrient acquisition [5, 11, 27, 28, 76, 85]. *B. conglomeratum* and *Glutamicibacter* sp. demonstrate exceptional CMC degradation, paralleling previous findings [63, 105]. Cellulase and amylase synthesis by *M. morganii* and *B. cereus*, and cellulase activity in *Klebsiella* and *Bacillus* strains, confirm prior research [16, 69].

Xylanase synthesis, highlighted by Sun and Shao [11, 91, 96], is echoed in BCW microbiota, with *B. conglomeratum* excelling. Pectin degradation is prevalent, as in other insect orders [5, 11, 85, 96]. Amylase production, common across plant parts [5], is crucial in various taxa [11, 27]. Lipase and protease activity is exhibited by Actinobacteria and Proteobacteria, paralleling findings in different orders [11, 31].

Our study reveals antibiotic impacts on BCW, resulting in reduced larval development and altered hemolymph markers. This echoes similar observations [97, 104].

The study probes BCW larvae’s interaction with microbial consortia. The BF group displays notable weight gain, accelerated pupation, and increased hemolymph biomarkers, paralleling similar trends [8, 73, 80, 89]. Our study underscores the power of cooperative microbial networks, enhancing larval development and biomarkers, akin to earlier research [37, 55]. These consortia’s success hinges on inter-member communication and division of labor [17, 46, 85, 99] .

In pest management, insect gut microbiota’s pesticide degradation prowess is pivotal. Actinobacteria and Firmicutes play a significant role, backed by substantial evidence [18, 21, 22, 70]. Numerous Actinobacteria genera, including *Streptomyces*, demonstrate pesticide degradation [18, 20, 22, 34, 86, 102]. Diverse orders host symbionts detoxifying pesticides, such as Lepidoptera species pesticides [20, 22, 34, 102]. γ-Proteobacteria like Enterobacteriaceae are key in degrading diverse pesticides, even in resistant populations [20, 22, 34, 102]. Additionally, Actinomycetes hold potential against pollutants (Mawang et al., 2021), producing enzymes like laccase and linamerase [42, 53].

A notable study highlight is *B. conglomeratum* and *Glutamicibacter* sp. chlorpyrifos degradation. This aligns with previous findings and reveals potential applications in biodegradation [75]. *B. conglomeratum* superior degradation underscores inter-population chemical communication [17]. Intriguingly, while coexisting harmoniously, antagonistic chlorpyrifos degradation interactions suggest *B. conglomeratum* promise in biodegradation efforts.

In summary, our study delves into the intricate dance of insect-microbe symbiosis, where coevolution shapes dynamic relationships. This partnership holds particular significance in addressing insect pests, offering insights into their ability to metabolize toxins and adapt their nutritional strategies. Our investigation into Lepidoptera’s microbiota, specifically the black cutworm (BCW), enriches our understanding of taxonomic composition and functional potential.

The taxonomic analysis of BCW gut microbiota confirms the prevalence of Proteobacteria, Actinobacteria, and Firmicutes, with Enterobacteriaceae family’s consistent dominance. This family’s role in polysaccharide degradation highlights its vital role, bridging developmental stages. Identifying genera like *Klebsiella*, *Enterobacter*, *Morganella*, and more underscores their presence and role [5, 16, 43, 50, 81, 88, 97, 103, 106, 107].

Functional enzyme potential within BCW gut microbiota shines light on its role in nutrient acquisition and digestion. Notable enzyme synthesis, including cellulases, xylanases, and pectinases, reaffirms the vital contribution of symbiotic partners in metabolic processes. Highlighting *B. conglomeratum* and *Glutamicibacter* sp. CMC degradation prowess aligns with prior studies and suggests promising candidates for biodegradation applications.

Exploring the impact of microbial consortia on BCW larvae emphasizes cooperative microbial networks’ significance, enhancing larval development and biomarkers. In the realm of pest management, the capability of insect gut microbiota to degrade pesticides is underscored, with Actinobacteria and Firmicutes playing pivotal roles. These findings contribute to potential bioremediation strategies and pave the way for sustainable pest management approaches.

Ultimately, this study’s narrative unfolds within the intricate web of life, offering glimpses into the microscopic choreography governing interactions between insects and microbes. With far-reaching implications spanning pest management, environmental protection, and beyond, the captivating realm of insect-microbe symbiosis remains a frontier of scientific exploration, promising novel insights and innovative solutions.

## Conclusion

Insect gut bacteria offer great potential for biotechnological applications. This study revealed that the gut bacterial communities in BCW larvae are capable of degrading various polysaccharides, including cellulose, xylan, pectin, and starch, and producing lipolytic and protease enzymes to aid BCW metabolism. Alterations in gut microbiota composition have a significant impact on the metabolism of BCW larvae. Furthermore, the gut bacteria have demonstrated the ability to degrade various pesticide classes, indicating their potential role in conferring pesticide resistance to the host. Therefore, gut bacteria could serve as a viable candidate for developing eco-friendly management strategies for pesticide bioremediation, such as in IPM programs. However, future research is required to explore combining bactericidal compounds with insecticides to eliminate symbiotic gut bacteria in insects. This study also highlights the superior polysaccharide digestion capabilities of bacterial consortia over individual isolates. To achieve synergistic bacterial degradation, it is crucial to ensure that individual isolates do not exhibit antagonistic interactions within a consortium. The findings of this study have potential applications in IPM programs as a new, environmentally friendly management strategy. Additionally, gut bacteria may have potential as probiotics for mass-production of insects in specialized laboratories.

## Data Availability

Data produced during this study are available from the corresponding author upon reasonable request.
